# Supplementary material for: Colonic In Vitro Model Assessment of the Prebiotic Potential of Bread Fortified with Polyphenols Rich Olive Fiber
Source: Nutrients. 2021 Feb 27;13(3):787. doi: 10.3390/nu13030787 (PMC7997273; doi:10.3390/nu13030787)
Supplement: Supplementary file 1 [file nutrients-13-00787-s001.pdf]

Supplementary Materials

Table S1. Primers pairs employed for PCR and qPCR reactions and quantifications.

Group	Target	Sequence 3′-5′	Bp	Reference
Eubacteria	V3-V4 16 S	Eub518R: ATTACCGCGGCTGCTGG<br>Eub338R: ACTCCTACGGGAGGCAG	147	Lane et al, 1991
Enterobacteriaceae	V3-V4 16 S	Enterobac-f: TGCCGTAACTTCGGGAG<br>Enterobac-r: TCAAGGACCAGTGTTCAG	450	Bartosh et al, 2004
Lactobacillales	V3-V4 16 S	F-Lac: GCAGCAGTAGGGAATCT<br>R-Lac: GCATTYCACCGCTACACA	340	Walter et al, 2001
Bifidobacteriaceae	RecA	RecAf: CGTYTCBCAGCCGGAYA<br>RecAr: CCARVGCRCCGGTCATC	220	Masco et al., 2006
E. coli	FtsZ	EcFtsZf: GGTATCCTGACCGTTGCT<br>EcFtszr: ATACCTCGGCCCAGAACT	250	Zhou e Helmstetter, 1994
Clostridiaceae	V3-V4 16 S	ClosIV-f: TTAACACAATAAGTWATC<br>ClosIV-r: ACCTTCCTCCGTTTTGTC	400	Goldberg et al., 2013

Table S2. Quantification of VOCs by SPME GC/MS related to prebiotic potential, employing an internal standard.

VOCs	mg/Kg ± SD		
Acetic acid	0.465	±	0.353
Propanoic acid	0.210	±	0.161
Butanoic acid	0.700	±	0.090
Propanoic acid, 2-methyl		traces	
Butanoic acid, 3-methyl	0.434	±	0.028
Pentanoic acid, 3-methyl	0.031	±	0.007
Pentanoic acid	0.689	±	0.122
Hexanoic acid	0.046	±	0.030
Heptanoic acid		traces	
Octanoic acid		traces	
Nonanoic acid		< 0.01	
n-Decanoic acid		< 0.01	
Indole	3.879	±	0.963
Skatole	0.408	±	0.063

*traces < 0.03 mg/Kg.

Table S3. Relative quantification (%) of significant species ($P < 0.05$) (cutoffs = 0.001).

Assigned OTUs	Baseline	FOS 24 h	Eco0% 24 h	Eco4% 24 h
Acidaminococcus;s__	0.0119 [a]	0.0000	17.0160 [b]	0.0000
Adlercreutzia;s__	0.0727 [a]	0.0165 [b]	0.0262 [b]	0.0259 [b]
Akkermansia;s__muciniphila	1.7751 [a]	2.6454 [a]	0.2026 [b]	1.8068 [a]
Anaerofilum;s__pentosovorans	0.0705 [a]	0.1974 [b]	0.2436 [b]	0.3538 [bc]
Anaerostipes;s__	0.2257 [a]	0.0000	0.0865 [b]	0.0155 [b]
*Bacteroides;*Other	1.2065 [a]	2.0981 [a]	7.3804 [b]	2.6273 [a]
Bacteroides;s__acidifaciens	0.1052 [a]	0.1691 [a]	0.4007 [bc]	0.7826 [c]
Bacteroides;s__caccae	0.6217	0.5140	0.6421	0.4564
Bacteroides;s__cellulosilyticus	0.2279 [a]	0.1487 [a]	7.2420 [b]	0.6635 [a]
Bacteroides;s__eggerthii	0.7270 [a]	0.2040 [b]	0.0228 [c]	0.0181 [c]
Bacteroides;s__fragilis	0.0250 [a]	1.0766 [b]	0.1252 [a]	1.2640 [b]
Bacteroides;s__massiliensis	0.4731 [a]	0.3567 [a]	0.3017 [a]	3.6914 [b]
Bacteroides;s__thetaiotaomicron	0.2702 [a]	0.2363 [a]	1.6632 [b]	2.5583
Bacteroides;s__uniformis	1.7512 [a]	0.3685 [b]	1.1851 [a]	3.8950 [c]
Bacteroides;s__ vulgatus	8.1140 [a]	2.1840 [b]	2.9667 [b]	3.9648 [b]
Bifidobacterium;s__adolescentis	3.0067 [a]	32.1257 [b]	2.1716 [a]	6.8630 [c]
Bifidobacterium;s__bifidum	0.7877 [a]	3.8160 [b]	0.7821 [a]	0.6489 [a]
Bifidobacterium;s__longum	1.3093 [a]	4.2386 [b]	2.2097 [b]	4.8900 [b]
Bilophila;s__wadsworthia	0.7862 [a]	0.0562 [b]	0.9735 [a]	0.3262 [a]
Blautia;s__	8.2039 [a]	0.1336 [b]	0.3358 [b]	0.1734 [b]
Blautia;s__obeum	2.5856 [a]	0.0526 [b]	0.2004 [b]	0.0742 [b]
Blautia;s__producta	0.2886 [a]	0.0000	0.0250 [b]	0.1363 [a]
Butyricimonas;s__	0.1562 [a]	0.0125 [b]	0.2163 [a]	0.1199 [a]
Citrobacter;s__freundii	0.7191 [a]	0.0010 [b]	0.0050 [b]	0.0289 [b]
Collinsella;s__aerofaciens	1.5353 [a]	2.2883 [a]	0.2425 [b]	0.4858 [b]
*Coprobacillaceae;*Other	0.2203 [a]	0.0099 [b]	0.0387 [b]	0.0242 [b]
Coprobacillus;s__cateniformis	0.0380 [a]	0.0138 [a]	0.5043 [b]	0.3727 [b]
Coprococcus;s__	6.9756 [a]	0.0270 [b]	0.2561 [b]	0.2563 [b]
*Coriobacteriaceae;*Other	1.0774 [a]	0.0592 [b]	0.2846 [b]	0.1484 [b]
Desulfovibrio;s__	0.5121 [a]	0.1053 [a]	0.8387 [b]	0.1631 [a]
Dialister;s__invisus	2.8362 [a]	0.0000	1.1813 [b]	0.9025 [b]
Dorea;s__formicigenerans	0.6478 [a]	0.3949 [a]	6.7692 [b]	1.3503 [a]
Enterococcus;s__durans	0.0174 [a]	5.4146 [b]	2.9246 [b]	7.5645 [b]
Escherichia;s__	0.4752 [a]	6.3164 [b]	9.6857 [b]	4.0286 [b]
Escherichia;s__albertii	0.6330 [a]	0.0230 [b]	1.0889 [a]	0.1430 [a]
Faecalibacterium;s__prausnitzii	8.7735 [a]	3.1281 [b]	0.0455 [c]	4.8725 [b]
Faecalibacterium;s__	0.2289 [a]	0.4607 [a]	0.1662 [a]	0.0216 [b]
Klebsiella;s__variicola	0.0043 [a]	0.0000	0.0057 [a]	0.0052 [a]
Lachnospira;s__	0.9570 [a]	0.7226 [a]	0.1366 [b]	0.1070 [b]
Lachnospira;s__pectinoschiza	0.1628 [a]	2.4107 [b]	0.0205 [c]	0.0069 [c]
*Lachnospiraceae;*Other	0.2919 [a]	0.0046 [b]	0.1617 [a]	0.6911 [a]
Lactobacillus;s__plantarum	0.0000	2.2958 [a]	0.0091 [b]	4.1990 [a]
Lactococcus;s__lactis	0.2930 [a]	1.1955 [a]	0.0023 [b]	0.0035 [b]
Megasphaera;s__elsdenii	1.8218 [a]	12.3391 [b]	9.0260 [b]	16.2232 [b]
Methanobrevibacter;s__smithii	0.6879 [a]	0.0526 [b]	0.2172 [a]	0.2502 [a]
Oscillospira;s__	2.1646 [a]	0.3225 [b]	0.8800 [b]	0.3857 [b]
Parabacteroides;s__	0.3917 [a]	0.0816 [a]	3.8454 [b]	2.2278 [b]
Parabacteroides;s__distasonis	4.4106 [a]	0.5278 [b]	3.0544 [a]	13.2508 [c]
Parabacteroides;s__merdae	0.1345	0.0000	0.0000	0.0000
Phascolarctobacterium;s__	0.0998 [a]	0.0224 [b]	0.0603 [a]	0.1415 [a]

*Porphyromonadaceae;*Other	0.0000	0.0724 ^{a}	0.6887 ^{b}	0.3201 ^{b}
Rikenella;s__microfusus	2.7842 ^{a}	0.1856 ^{b}	0.8504 ^{b}	1.2468 ^{b}
Roseburia;s__	2.6909 ^{a}	0.1316 ^{b}	0.0273 ^{b}	0.0544 ^{b}
Roseburia;s__faecis	0.1367 ^{a}	2.3558 ^{b}	0.0137 ^{c}	0.0017 ^{d}
*Ruminococcus;*Other	10.6419 ^{a}	1.0395 ^{b}	0.5007 ^{b}	0.0000
Ruminococcus;s__	2.8015 ^{a}	0.2896 ^{b}	2.2461 ^{a}	0.4823 ^{b}
Ruminococcus;s__callidus	1.0102 ^{a}	0.0000	0.0011 ^{b}	0.0035 ^{b}
Ruminococcus;s__gnavus	3.1470 ^{a}	0.1777 ^{b}	0.1776 ^{b}	0.2457 ^{b}
Ruminococcus;s__torques	0.7009 ^{a}	0.0000	0.0091 ^{b}	0.0052 ^{b}
Slackia;s__isoflavoniconvertens	0.2040 ^{a}	0.0243 ^{b}	0.3700 ^{a}	0.1320 ^{a}
Sutterella;s__	0.4720 ^{a}	0.6199 ^{a}	1.1236 ^{b}	0.3702 ^{a}
Tepidibacter;s__	1.4843 ^{a}	0.0816 ^{b}	0.1685 ^{b}	0.0682 ^{b}

^{abc}Different letters within a line indicate statistical significance by t-student test ($P < 0.05$).

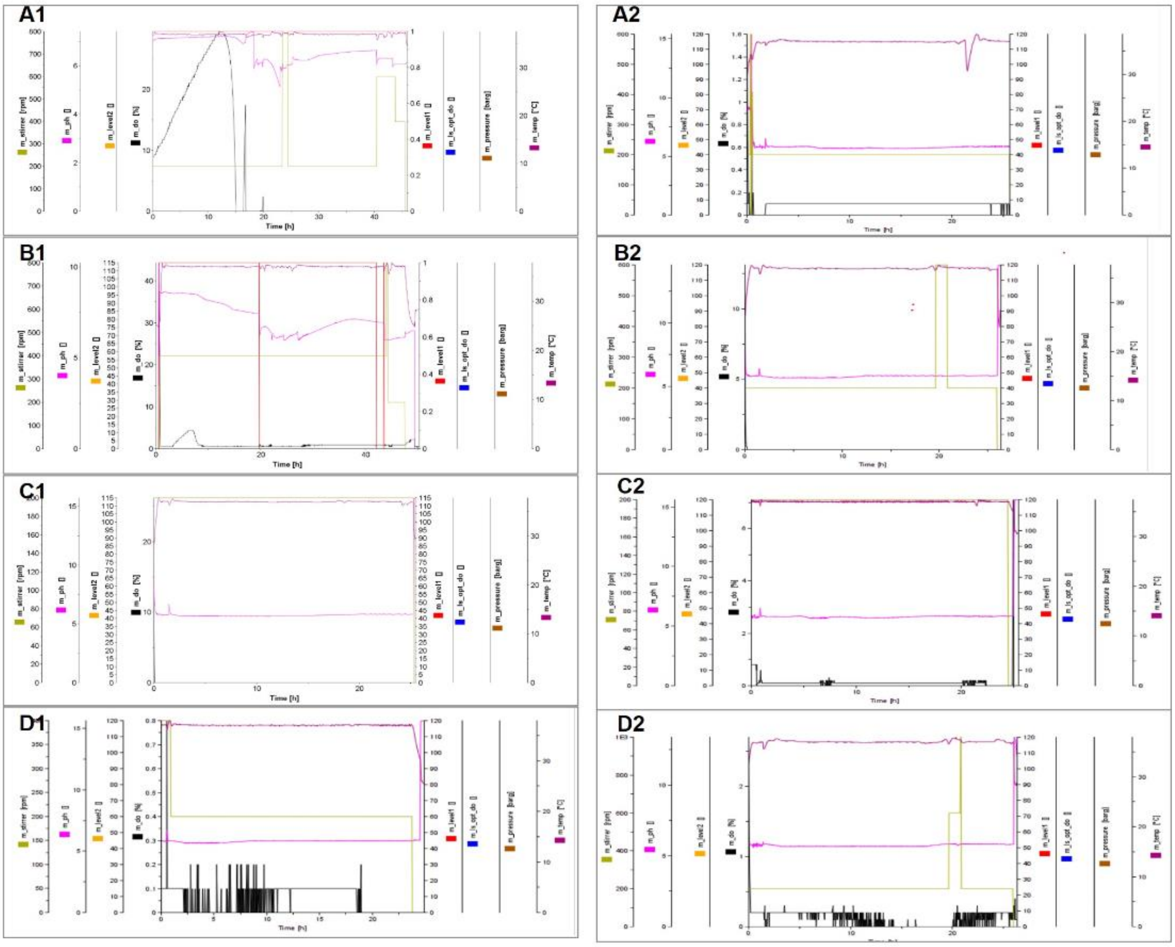

Figure S1. Plots of parameters trends of bioreactors, during colonic fermentations

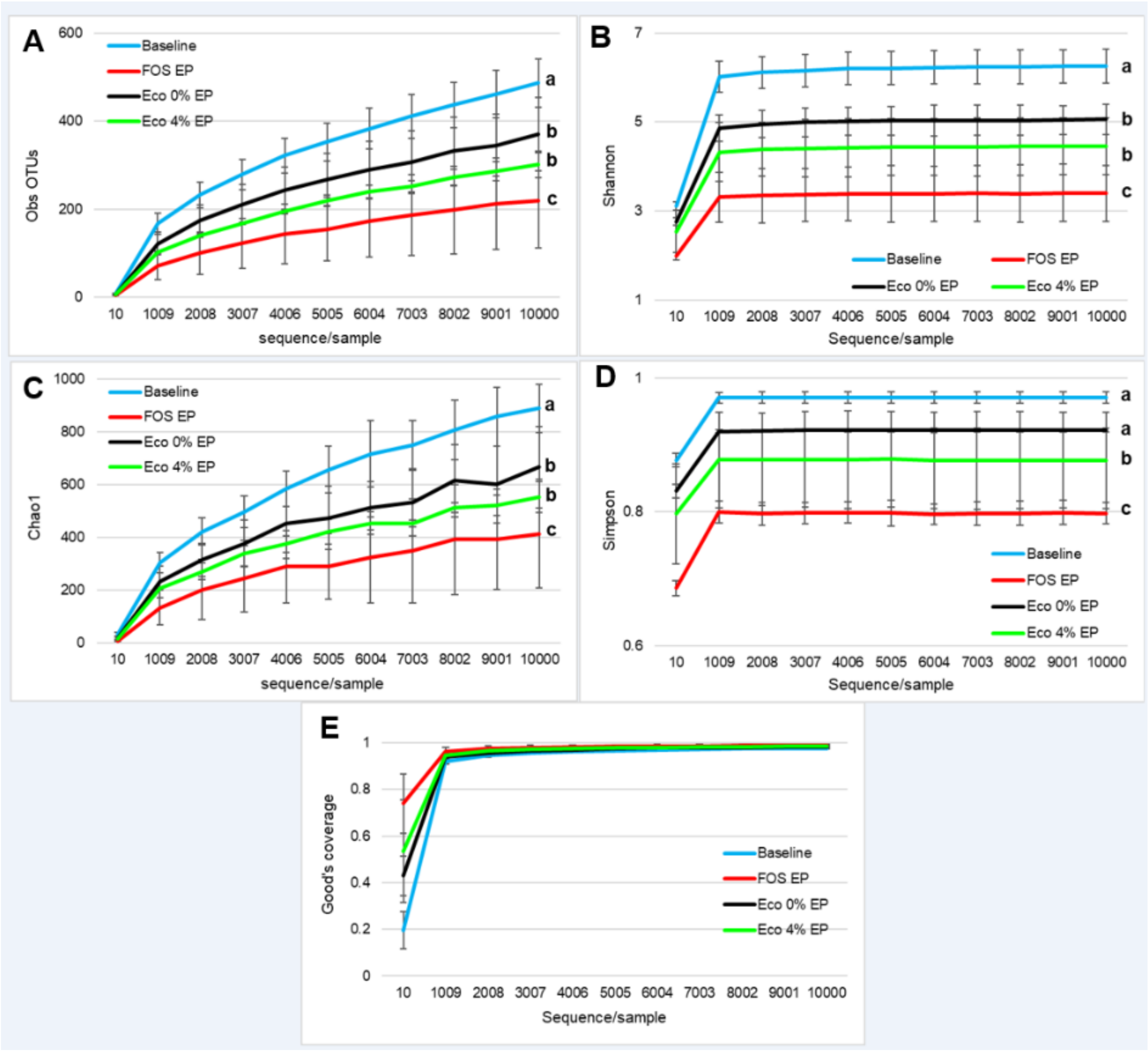

Figure S2. Plots of Alpha Diversity indices

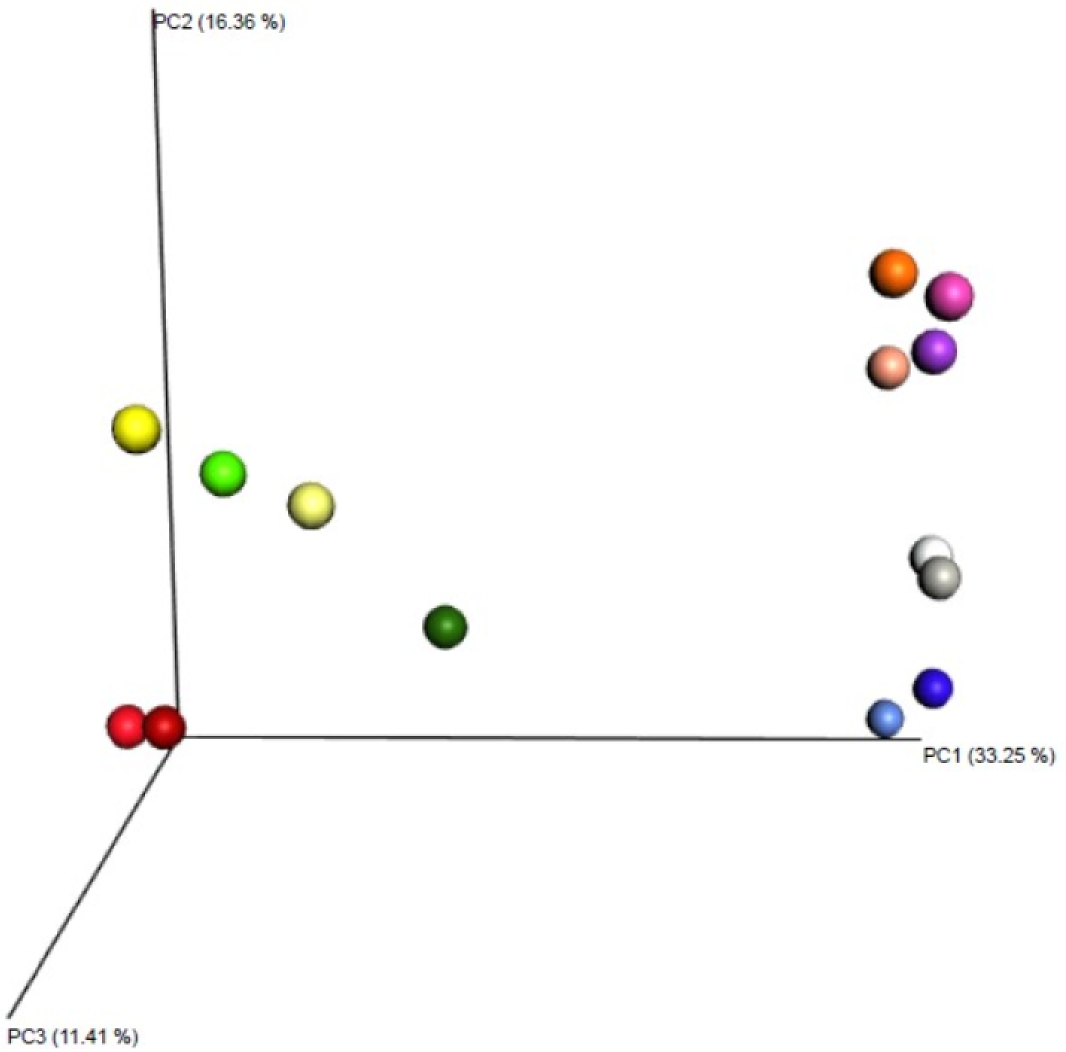

Figure S3. Bray Curtis PCoA of Beta Diversity

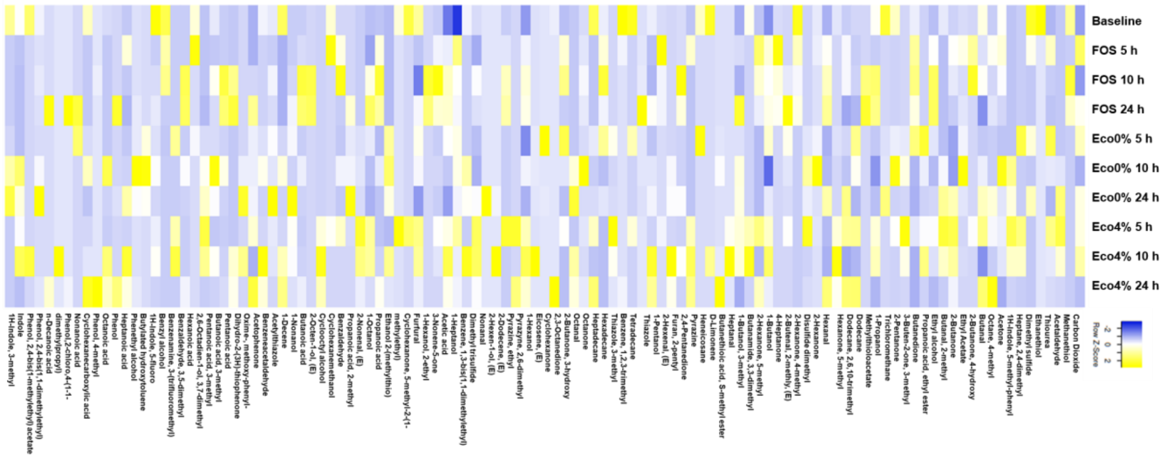

Figure S4. Quantification heatmap of total VOCs

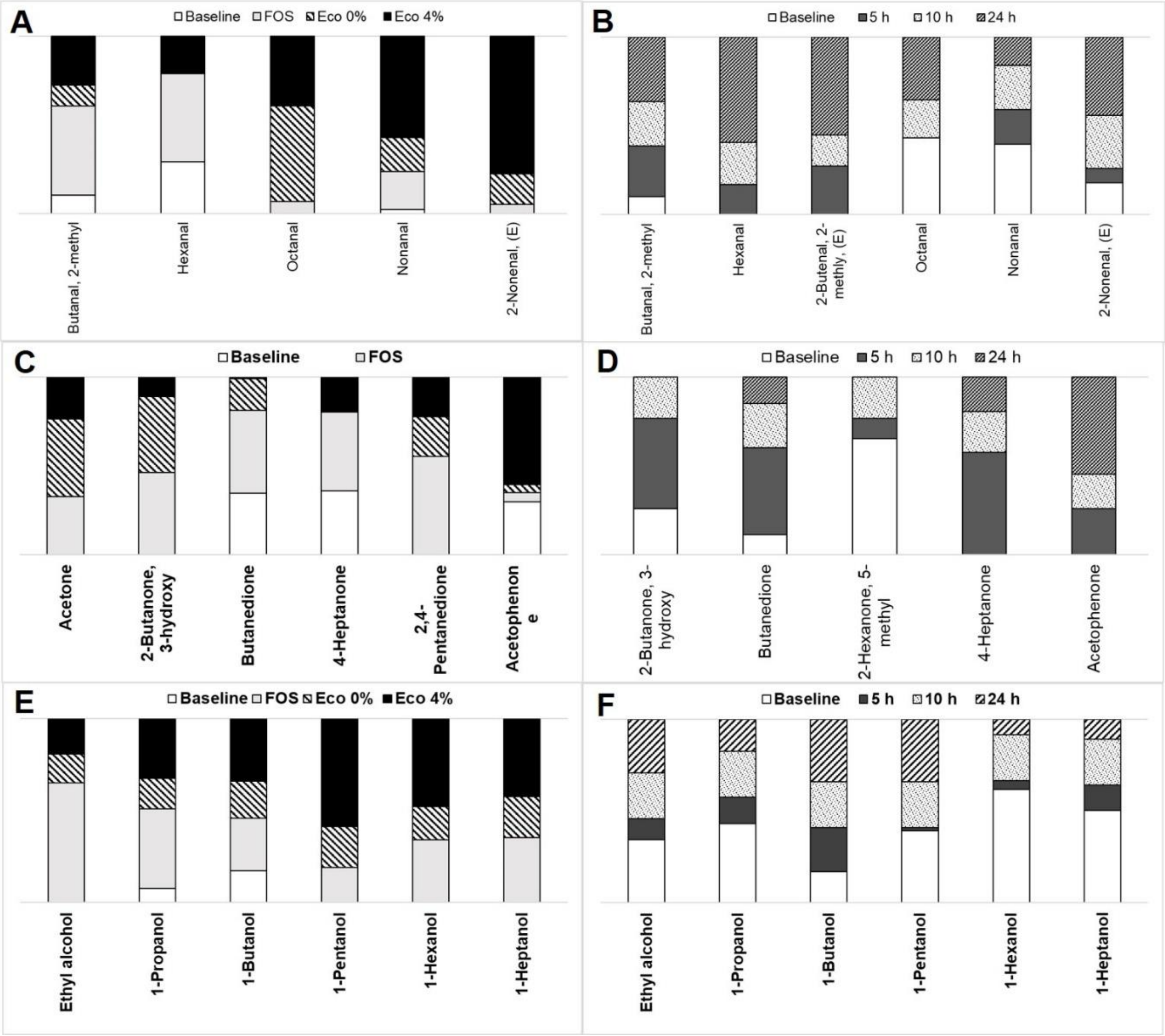

Figure S5. MANOVA plots by categorical descriptors for the volatilome

Spearman Rank Order Correlations (Spreadsheet34)
MD pairwise deleted
Marked correlations are significant at p <,05000

Variable	Acetic acid	Propanoic acid	Butanoic acid	Propanoic acid, 2-methyl	Butanoic acid, 3-methyl	Pentanoic acid, 3-methyl	Pentanoic acid	Hexanoic acid	Heptanoic acid	Octanoic acid	Nonanoic acid	n-Decanoic acid	Indole	1H-Indole, 3-methyl
Bifidobacterium;s__adolescentis	0,535714	0,689286	0,707143	0,157143	0,157143	-0,542857	0,564286	0,564286	0,648750	-0,150000	0,697051	0,564286	0,157143	-0,560714
Bifidobacterium;s__bifidum	-0,721429	-0,550000	-0,542857	0,596429	0,596429	0,778571	-0,725000	-0,725000	-0,591402	-0,582143	-0,555854	-0,725000	0,596429	0,539286
Bifidobacterium;s__longum	-0,882143	-0,782143	-0,775000	0,364286	0,364286	0,903571	-0,885714	-0,885714	-0,806457	-0,385714	-0,788204	-0,885714	0,364286	0,675000
Bacteroides;s__caccae	-0,860714	-0,710714	-0,689286	0,260714	0,260714	0,846429	-0,832143	-0,832143	-0,763446	-0,260714	-0,700626	-0,832143	0,260714	0,517857
Bacteroides;s__cellulosilyticus	-0,500000	-0,464286	-0,457143	-0,400000	-0,400000	0,435714	-0,457143	-0,457143	-0,483874	0,435714	-0,453977	-0,457143	-0,400000	0,132143
Bacteroides;s__vulgatus	-0,675000	-0,717857	-0,710714	0,110714	0,110714	0,646429	-0,671429	-0,671429	-0,745525	-0,010714	-0,702413	-0,671429	0,110714	0,360714
Parabacteroides;s__distasonis	-0,625000	-0,475000	-0,467857	-0,214286	-0,214286	0,603571	-0,582143	-0,582143	-0,505380	0,221429	-0,478999	-0,582143	-0,214286	0,264286
Rikenella;s__microfusus	-0,489286	-0,635714	-0,639286	-0,260714	-0,260714	0,432143	-0,500000	-0,500000	-0,620076	0,303571	-0,627346	-0,500000	-0,260714	0,271429
Enterococcus;s__durans	-0,310714	-0,610714	-0,646429	-0,192857	-0,192857	0,317857	-0,382143	-0,382143	-0,526885	0,207143	-0,639857	-0,382143	-0,192857	0,432143
Blautia;s__	-0,575000	-0,789286	-0,803571	-0,007143	-0,007143	0,553571	-0,617857	-0,617857	-0,752693	0,042857	-0,793566	-0,617857	-0,007143	0,507143
Dorea;s__formicigenerans	-0,578571	-0,335714	-0,328571	0,489286	0,489286	0,635714	-0,564286	-0,564286	-0,376347	-0,510714	-0,350313	-0,564286	0,489286	0,417857
Lachnospira;s__pectinoschiza	0,760714	0,503571	0,496429	0,021429	0,021429	-0,789286	0,710714	0,710714	0,544806	0,014286	0,504022	0,710714	0,021429	-0,492857
Ruminococcus;s__	-0,707143	-0,471429	-0,450000	0,485714	0,485714	0,728571	-0,678571	-0,678571	-0,541222	-0,464286	-0,461126	-0,678571	0,485714	0,389286
Faecalibacterium;s__prausnitzii	0,132143	-0,203571	-0,232143	-0,225000	-0,225000	-0,160714	0,078571	0,078571	-0,136202	0,310714	-0,212690	0,078571	-0,225000	-0,021429
Oscillospira;s__	-0,817857	-0,957143	-0,960714	0,435714	0,435714	0,825000	-0,875000	-0,875000	-0,942658	-0,428571	-0,957999	-0,875000	0,435714	0,750000
Dialister;s__invisus	-0,553571	-0,535714	-0,510714	0,885714	0,885714	0,560714	-0,582143	-0,582143	-0,591402	-0,850000	-0,507596	-0,582143	0,885714	0,432143
Megasphaera;s__elsdenii	0,457143	0,557143	0,564286	0,364286	0,364286	-0,435714	0,453571	0,453571	0,537638	-0,385714	0,552279	0,453571	0,364286	-0,342857
Collinsella;s__aerofaciens	0,850000	0,703571	0,700000	-0,260714	-0,260714	-0,907143	0,839286	0,839286	0,724019	0,303571	0,713137	0,839286	-0,260714	-0,746429
Slackia;s__isoflavoniconvertens	-0,907143	-0,839286	-0,828571	0,439286	0,439286	0,921429	-0,917857	-0,917857	-0,867389	-0,453571	-0,838249	-0,917857	0,439286	0,700000
Sutterella;s__	-0,392857	-0,464286	-0,457143	0,939286	0,939286	0,435714	-0,457143	-0,457143	-0,483874	-0,903571	-0,453977	-0,457143	0,939286	0,453571
Bilophila;s__wadsworthia	-0,867857	-0,703571	-0,682143	0,253571	0,253571	0,853571	-0,839286	-0,839286	-0,756277	-0,267857	-0,693477	-0,839286	0,253571	0,525000
Citrobacter;s__freundii	-0,285714	-0,553571	-0,592857	-0,267857	-0,267857	0,300000	-0,350000	-0,350000	-0,465953	0,275000	-0,589813	-0,350000	-0,267857	0,407143
Escherichia;s__	0,582143	0,446429	0,425000	-0,857143	-0,857143	-0,646429	0,596429	0,596429	0,501795	0,864286	0,428955	0,596429	-0,857143	-0,557143
Escherichia;s__albertii	0,582143	0,446429	0,425000	-0,857143	-0,857143	-0,646429	0,596429	0,596429	0,501795	0,864286	0,428955	0,596429	-0,857143	-0,557143
Akkermansia;s__muciniphila	0,096429	-0,039286	-0,046429	-0,675000	-0,675000	-0,189286	0,117857	0,117857	-0,014337	0,725000	-0,032172	0,117857	-0,675000	-0,282143

Figure S6. Significance of Spearman rank correlations
